# Invasive Fungal Disease Associated With Targeted Agents for Acute Myeloid Leukaemia: A Systematic Review

**DOI:** 10.1002/jha2.1105

**Published:** 2025-03-10

**Authors:** Samir Agrawal, Anjaneya Bapat, Christopher P. Eades, Shreyans Gandhi

**Affiliations:** ^1^ Queen Mary University of London London UK; ^2^ St. Bartholomew's Hospital, Bart's Health NHS Trust London UK; ^3^ Department of Infection King's College Hospital NHS Foundation Trust London UK; ^4^ Department of Infectious Diseases Manchester University NHS Foundation Trust Manchester UK; ^5^ Mycology Reference Centre Manchester (MRCM), ECMM Centre of Excellence Wythenshawe Hospital Manchester UK; ^6^ Department of Haematology King's College Hospital NHS Foundation Trust London UK

**Keywords:** acute myeloid leukaemia, haematological malignancy, invasive fungal disease, leukaemia, targeted agent, targeted therapy

## Abstract

**Objective:**

To examine the incidence of invasive fungal disease (IFD) in patients receiving targeted agents for acute myeloid leukaemia (AML).

**Methods:**

Literature for this systematic review was identified through a PubMed search in April 2024, using AML, IFD and targeted therapy terms. The following filters were applied: published in the last 10 years and published in English.

**Results:**

The PubMed search yielded 54 results, of which 16 were deemed relevant for inclusion. Four additional references were identified through manual searches. The majority of publications focused on the incidence of IFD during treatment with targeted agents; the remainder focused on the efficacy of targeted treatments and reported IFD as an adverse event. Most publications were retrospective analyses. Prophylaxis use and agents differed across studies. In several studies, IFD incidence was above the 8% threshold identified for anti‐mould prophylaxis. *Aspergillus* was the most commonly reported pathogen, and most IFD cases occurred in the lungs.

**Conclusions:**

IFD is relatively common among patients with AML receiving targeted therapies, despite the use of prophylaxis. Prospective studies with detailed IFD reporting, together with large epidemiological studies, are required to better understand the risk factors for, and incidence and nature of IFD in this patient population.

## 1 Introduction

Invasive fungal disease (IFD) presents a significant challenge to the care of patients with haematological malignancies, affecting up to 11% of patients [1, 2]. Overall rates of IFD in this patient population are decreasing, largely due to advances in diagnosis and prophylaxis [3, 4]. However, morbidity and mortality of such infections remain high, with mortality rates ranging from 29% to 90% [1, 3]. Compared with the wider haemato‐oncology patient population, individuals with acute myeloid leukaemia (AML) have an increased risk of IFD [2]. Further, certain conditions are associated with high risk of IFD independent of the underlying disease, namely, presence of neutropenia, use of high‐dose steroids, relapsed/refractory (r/r) disease and a previous history of IFD [2].

As of September 2024, nine targeted agents are approved by the US Food and Drug Administration (FDA) for AML [5]. Of these, seven are also approved in Europe [6, 7, 8, 9, 10, 11, 12]. Indications for approved agents are summarised in Table 1. These agents include FMS‐like tyrosine kinase 3 (FLT3) and Hedgehog pathway inhibitors, both of which disrupt discrete signalling pathways crucial for leukaemia cell growth, proliferation and survival [13]; isocitrate dehydrogenase (IDH) inhibitors, which block mutant IDH enzyme activity in leukaemia cells and prevent the production of *R*‐2‐hydroxyglutarate, a mediator of the cells’ oncogenic potential [14]; monoclonal antibodies against CD33 [5] and B‐cell lymphoma 2 (BCL‐2) inhibitors, which inhibit the antiapoptotic BCL‐2 protein that is overexpressed in malignant cells [4].

**TABLE 1:** Overview of FDA‐ and/or EMA‐approved targeted agents used to treat AML.

Class	Agent	FDA‐approved indication	EMA‐approved indication
FLT3 inhibitor	Midostaurin (Rydapt) [11, 15]	Adults with newly diagnosed AML; *FLT3* mutation‐positive; in combination with standard cytarabine and daunorubicin induction and cytarabine consolidation chemotherapy	Adults with newly diagnosed AML; *FLT3* mutation‐positive; in combination with standard daunorubicin and cytarabine induction and high‐dose cytarabine consolidation chemotherapy, and for patients in complete response, followed by single‐agent maintenance therapy
	Quizartinib (Vanflyta) [12, 16]	Adults with newly diagnosed AML; *FLT3–ITD* mutation‐positive; in combination with standard cytarabine and anthracycline induction and cytarabine consolidation and as maintenance monotherapy, following consolidation chemotherapy	Adults with newly diagnosed AML; *FLT3–ITD* mutation‐positive; in combination with standard cytarabine and anthracycline induction and standard cytarabine consolidation chemotherapy, followed by single‐agent maintenance therapy
	Gilteritinib (Xosparta) [8, 17]	Adults with r/r AML; *FLT3* mutation‐positive	Adults with r/r AML; *FLT3* mutation‐positive
IDH inhibitor	Ivosidenib (Tibsovo) [7, 18]	Adults with r/r AML; susceptible *IDH‐1* mutation‐positive Adults with newly diagnosed AML, aged ≥ 75 years or with comorbidities that preclude use of intensive induction chemotherapy; susceptible *IDH‐1* mutation‐positive; in combination with azacitidine or as monotherapy	Adults with newly diagnosed AML who are ineligible for standard induction chemotherapy; *IDH‐1* R132 mutation‐positive; in combination with azacitidine
	Olutasidenib (Rezlidhia) [19]	Adults with r/r AML; susceptible *IDH‐1* mutation‐positive	Not currently licensed for use in the EU
	Enasidenib (Idhifa) [20, 21]	Adults with r/r AML; *IDH‐2* mutation‐positive	Application for EU licencing withdrawn
CD33 antibody–drug conjugate	Gemtuzumab ozogamicin (Mylotarg) [10, 22]	Adult and paediatric patients aged ≥ 1 month with newly diagnosed AML; CD33‐positive Adult and paediatric patients aged ≥ 2 years with r/r AML; CD33‐positive	Patients aged ≥ 15 years with de novo, untreated AML; CD33‐positive; except APL; in combination with daunorubicin and cytarabine
BCL‐2 inhibitor	Venetoclax (US: Venclexta; EU: Venclyxto) [6, 23]	Adults with newly diagnosed AML, aged ≥ 75 years or with comorbidities that preclude use of intensive induction chemotherapy; in combination with azacitidine, or decitabine, or low‐dose cytarabine	Adults with newly diagnosed AML who are ineligible for intensive chemotherapy; in combination with a hypomethylating agent
Hedgehog pathway inhibitor	Glasdegib (Daurismo) [9, 24]	Adults with newly diagnosed AML, aged ≥ 75 years or with comorbidities that preclude use of intensive induction chemotherapy; in combination with low‐dose cytarabine	Adults with newly diagnosed de novo or secondary AML who are ineligible for standard induction chemotherapy; in combination with low‐dose cytarabine

Abbreviations: AML, acute myeloid leukaemia; APL, acute promyelocytic leukaemia; BCL‐2, B‐cell lymphoma 2; CD, cluster of differentiation; EMA, European Medicines Agency; EU, European Union; FDA, Food and Drug Administration; FLT3, FMS‐like tyrosine kinase 3; HSCT, haematopoietic stem cell transplantation; IDH, isocitrate dehydrogenase; ITD, internal tandem duplication; r/r, relapsed/refractory.

Determining how much of the IFD risk is due to the specific targeted treatment itself is difficult, given that patients with AML frequently receive combination therapy, and have often previously received cytotoxic chemotherapy (for which IFD prophylaxis recommendations and risk vary) or another targeted therapy. Furthermore, patients with AML have immune defects related to the disease itself that can significantly increase their risk of IFD [4]. In healthy individuals, neutrophils act as one of the first lines of defence against the development of IFD, primarily via phagocytosis and the direct killing of pathogens [25]. For patients with AML, this response is impaired, with patients tending to exhibit baseline neutrophil dysfunction and higher rates of neutropenia, which puts them at an increased risk of IFD [4]. The targeted inhibition of BCL‐2 for the treatment of AML has been linked to an increased risk of neutropenia, with a presumed mechanism of on‐target inhibition of BCL‐2 in neutrophil precursor cells [26]. In one trial, ≥ Grade 3 neutropenia occurred in 42% of patients treated with the BCL‐2 inhibitor venetoclax (VEN) in combination with azacitidine versus 28% of those who received azacitidine and placebo [27]. Genetic factors may also play a role in an individual's predisposition to IFD; mutation of the *FLT3* gene – common in patients with AML [28] – may potentially result in an intrinsic predisposition to IFD or a reduced susceptibility to antifungal prophylaxis. FLT3 is an immune‐enhancing molecule; thus, inhibition of FLT3 can reduce the immune‐enhancing effect; in preclinical studies, FLT3 inhibitors inhibited production of Type I interferons and impaired the development of dendritic cells – two major contributors to the healthy immune response [29].

With the above considerations in mind, the objective of this systematic review is to examine the incidence of IFD in patients receiving targeted agents for AML.

## 2 Antifungal Prophylaxis Recommendations in AML

An overview of recommendations on antifungal prophylaxis in patients receiving targeted agents for AML is given in Table 2.

**TABLE 2:** Key guideline recommendations on antifungal prophylaxis and IFD treatment in patients receiving targeted agents^a^ for the treatment of AML.

Reference	Society/organisation	Country/region	Type of guidance	Patient population	Treatment	Infectious diseases covered	Key antifungal prophylaxis recommendations for patients with AML
Stemler et al. [30]	European Hematology Association (EHA)	Europe	Systematic review and expert consensus recommendation	Adults with AML	Targeted therapies	IFD	Antifungal prophylaxis is recommended with moderate strength in most settings and strongly recommended if the novel AML agent is administered in combination with intensive induction chemotherapy *FLT3 inhibitor* *Midostaurin*: Conditional for use; triazole prophylaxis is recommended in patients at a high risk of IFD, preferably with posaconazole. Conditional recommendation in favour of prophylaxis in patients at low risk but are neutropenic or have a history of IFD *Quizartinib*: Conditional for use; strong recommendation for triazoles during remission‐induction treatment with a dose reduction of quizartinib; no recommendation for antifungal prophylaxis when used as a monotherapy *Gilteritinib*: Context‐dependent recommendation either for and against use; triazole prophylaxis is recommended in cases where patients are at high risk of developing IFD *IDH inhibitor* *Ivosidenib*: Context‐dependent recommendation either for or against use; strong recommendation for antifungal prophylaxis when receiving in combination with another therapy. Recommendation against antifungal prophylaxis when used as a monotherapy *Enasidenib*: No recommendations *CD33 antibody–drug conjugate* *Gemtuzumab ozogamicin*: Conditional for use; strong recommendation for triazoles during remission‐induction treatment *BCL‐2 inhibitor* *Venetoclax*: Conditional for use; antifungal prophylaxis is recommended for patients treated with venetoclax in combination with an HMA and at high risk of IFD; preference for triazoles, dose adaptations may be required when given in combination with posaconazole or voriconazole *Hedgehog pathway inhibitor* *Glasdegib*: Conditional against use
Stemler et al. [31]	Infectious Diseases Working Party (AGIHO) of the German Society for Haematology and Medical Oncology (DGHO)	Germany	Working party recommendations	Haematological malignancies, mainly focused on AML and MDS	Targeted therapies	IFD	Prophylaxis should be administered preferably with mould‐active azoles or an echinocandin, whereby posaconazole remains the drug of choice due to its efficacy and readily absorbable oral tablet formulation Patients with persistent neutropenia due to active underlying malignant disease and thus an increased risk of IFD may also benefit from antifungal prophylaxis *FLT3 inhibitor* *Midostaurin*: If indicated, use triazole antifungal prophylaxis without dose adjustment *Quizartinib*: If indicated, use triazole antifungal prophylaxis without dose adjustment; reduce quizartinib dose when given in combination with posaconazole or voriconazole to prevent toxicity *Gilteritinib*: use triazole antifungal prophylaxis without dose adjustment *IDH inhibitor* *Ivosidenib*: If indicated, use triazole antifungal prophylaxis without dose adjustment; reduce ivosidenib dose when given in combination with posaconazole or voriconazole to prevent toxicity *BCL‐2 inhibitor* *Venetoclax*: use triazole antifungal prophylaxis; dose of venetoclax should be reduced by at least 75% when administered concomitantly with posaconazole or voriconazole or by 50% when administered with fluconazole or isavuconazole
Teh et al. [32]	Australasian Antifungal Guidelines Steering Committee	Australasia	Steering committee guidelines	Haematological malignancies and stem cell transplant recipients	Targeted therapies	IFD	Patients in the induction/re‐induction stage of AML treatment are considered high‐risk for IFD, regardless of agent, and antifungal prophylaxis is recommended. When using a strong CYP3A4 inhibitor, such as posaconazole, itraconazole or voriconazole, in combination with novel targeted therapies that are major CYP3A4 substrates, such as venetoclax, dose reductions of up to 75% of the targeted agents are indicated
Maertens et al. [33]	European Conference on Infections in Leukaemia (ECIL)	Europe	Society guidelines	Haematological malignancies and stem cell transplant recipients	n/a	IFD	No specific recommendations given for prophylaxis with targeted therapies. Recommendations for traditional chemotherapy are given below Azoles are considered the first choice for primary antifungal prophylaxis for patients receiving intensive remission‐induction chemotherapy for AML or MDS. Posaconazole is the drug of choice Posaconazole remains the drug of choice when the incidence of invasive mould diseases exceeds 8% Primary antifungal prophylaxis not recommended beyond remission‐induction chemotherapy, unless patients are to undergo re‐induction chemotherapy or intensified consolidation therapy
Maertens et al. [34]	European Conference on Infections in Leukaemia (ECIL)	Europe	Society guidelines	Haematological malignancies and stem cell transplant recipients	n/a	PJP	No specific recommendations given for prophylaxis with targeted therapies in adults with AML
Taplitz et al. [35]	American Society of Clinical Oncology (ASCO)/Infectious Diseases Society of America (IDSA)	USA	Systematic review and expert consensus recommendation	Patients receiving treatment of cancer as inpatients or outpatients who are experiencing immune suppression or increased susceptibility to infection	n/a	Various	No specific recommendations given for prophylaxis with targeted therapies Antifungal prophylaxis with an oral triazole or parenteral echinocandin is recommended for patients who are at risk for profound, protracted neutropenia, such as most patients with AML or HSCT; a mould‐active triazole is recommended when the risk of invasive aspergillosis is > 6%, that is, in patients with AML/MDS

Abbreviations: AML, acute myeloid leukaemia; BCL‐2, B‐cell lymphoma 2; CD, cluster of differentiation; CYP3A4, cytochrome P450 3A4; FDA, Food and Drug Administration; FLT3, FMS‐like tyrosine kinase 3; HMA, hypomethylating agent; HSCT, hematopoietic stem cell transplantation; IDH, isocitrate dehydrogenase; IFD, invasive fungal disease; MDS, myelodysplastic syndrome.

^a^ Includes recommendations for all FDA‐approved targeted agents for the treatment of AML, as per the American Cancer Society website [5]. If an agent is not included in the table, it is because no specific recommendations were provided in the guidelines.

The European Hematology Association (EHA) has published recommendations on antifungal prophylaxis specifically in patients with AML receiving targeted therapies (Table 2) [30]. Antifungal prophylaxis is recommended ‘with moderate strength in most settings and strongly recommended if the novel AML agent is administered in combination with intensive induction chemotherapy' [30]. Guidance on which antifungal to use is not given.

The Infectious Diseases Working Party (AGIHO) of the German Society for Haematology and Medical Oncology (DGHO) has published guidelines on primary antifungal prophylaxis (PAP) in haematological malignancies, mainly focused on AML and myelodysplastic syndrome (MDS). Triazole antifungal prophylaxis (posaconazole [PCZ] being the drug of choice) is recommended for patients receiving VEN, gilteritinib, midostaurin, quizartinib and ivosidenib. To prevent and avoid toxicity, targeted therapy dose adjustment is recommended for VEN, quizartinib and ivosidenib when used in combination with PCZ or voriconazole (VCZ). VEN dose adjustment is also recommended when used with fluconazole (FCZ) or isavuconazole (ISAV) (Table 2) [31].

Guidelines from the Australasian Antifungal Guidelines Steering Committee recommend that, in haematological malignancy, antifungal prophylaxis be considered according to individual patient risk (Table 2) [32].

Several other guidelines have been published on antifungal/antimicrobial prophylaxis in haematological malignancy, though not specifically in patients receiving targeted agents for AML (Table 2) [33, 34, 35, 36].

Neither the American Society of Haematology (ASH) nor the British Society of Haematology (BSH) has published guidelines on antifungal prophylaxis in patients receiving targeted agents for AML. In the absence of a national guideline in the United Kingdom, many National Health Service trusts have their own antimicrobial protocols, leading to wide variations in practice.

## 3 Methods

Literature for inclusion were identified through a PubMed search in June 2024, combining AML terms with IFD terms and targeted therapy terms. The full list of search terms is shown in Table S1.

The IFD terms included common species (including a range of moulds, yeasts and dimorphic fungi, as well as *Pneumocystis jiroveci* pneumonia [PJP]) along with rare yeast species (as defined by the European Confederation for Medical Mycology [ECMM]/International Society for Human and Animal Mycology [ISHAM] and the American Society for Microbiology [ASM] [37]). Additional terms were included to reflect changes in nomenclature for common *Candida* species [38].

Targeted agent terms were compiled using the list of FDA‐approved targeted agents on the American Cancer Society website for AML [5].

Two filters were applied to the PubMed search: published in the last 10 years and published in English. Case reports and case series were initially included in the search but were removed manually once it was apparent that there was sufficient evidence from studies. Studies in paediatric patients were also excluded due to the different clinical and radiological presentation of IFD in this population compared with adults [39].

## 4 Results

The PubMed search generated 54 results. Of these, 16 were deemed relevant. The main reasons for excluding papers were as follows: case reports/case series (*n* = 8), pharmacokinetic study with no IFD data (*n* = 7) and reviews (*n* = 6). No duplicates were identified. The majority of publications (*n* = 14) focused on the incidence of IFD (or infections overall) during treatment with targeted agents. The remainder focused on the efficacy of targeted treatments and reported IFD as an adverse event (AE). Results are therefore reported below according to these two publication types. Most of the studies identified were retrospective in design.

An additional four publications were identified by manually searching reference lists of relevant reviews. Three of these publications were efficacy studies in which IFD was reported as an AE in the main text but not in the publication title or abstract.

**FIGURE 1:**
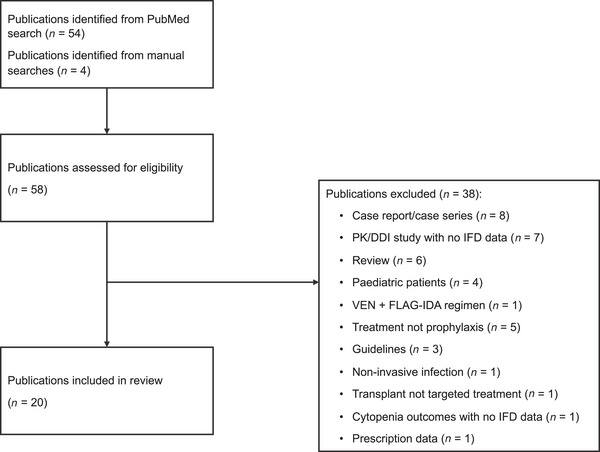
PRISMA flowchart showing papers excluded at each stage. DDI, drug–drug interaction; IFD, invasive fungal disease; PK, pharmacokinetic; VEN + FLAG‐IDA, venetoclax + fludarabine, high‐dose cytarabine and idarubicin.

A PRISMA flow chart describing the papers included/excluded at each stage is shown in Figure 1. The study design and key findings for papers included in the review are summarised in Table 3.

**TABLE 3:** Summary of study designs and key findings for the 20 papers included in the review.^a^

Reference	Study design	Country	IFD/efficacy focus	*N*	Patient population	Targeted agent	Prophylaxis	IFD criteria	Key findings
Cattaneo et al. [29]	Multicentre Observational	Italy	IFD	114	AML with *FLT3* mutation	Midostaurin (+ chemotherapy) for induction, re‐induction or consolidation therapy	Optional: given in 106/114 patients (93%) and 10/12 patients (83%) during induction and re‐induction, respectively; given to patients during 73/160 courses of consolidation chemotherapy (4.4%) Most commonly PCZ (48.2%); echinocandin (50%) and echinocandin (16.9%) during induction, re‐induction and consolidation therapy, respectively	EORTC/MSG	Proven, probable or possible IFD was reported in 23/114 patients (20.2%) and 4/12 patients (33.3%) during induction and re‐induction therapy, respectively. During consolidation therapy, seven cases of IFD were reported across a total of 160 courses (4.4%), all but one case occurred during the first consolidation course Most common pathogens were *Aspergillus* and *Candida* spp. in all three stages Most commonly affected site was the lung in all three stages IFD occurred with prophylaxis in 21/23, 4/4 and 0/7 patients during induction, re‐induction and consolidation therapy, respectively
Bose et al. [40]	Single‐centre Prospective Phase 2	USA	IFD	65	AML (95%)/MDS (5%)	VEN and/or FLT3 inhibitor (sorafenib^b^/midostaurin; *n* = 32)	ISAV	EORTC/MSG	Probable or possible IFD in five patients who received VEN‐based remission‐induction chemotherapy Probable pulmonary aspergillosis (*n* = 1), possible fungal pneumonia (*n* = 4) No IFD in patients receiving FLT3 inhibitor‐based remission‐induction chemotherapy
Candoni et al. [41]	Multicentre Prospective	Italy	IFD	230	Treatment‐naïve AML	VEN + HMA (DEC/AZA; *n* = 132)	Optional; anti‐mould prophylaxis given in 17/62 patients (27%) who developed pneumonia overall	Microbiologically or radiologically documented	67 pneumonia episodes (*n* = 62) 18/67 episodes (27%) had a fungal aetiology 10/62 patients with pneumonia died due to pneumonia
Phoompoung et al. [42]	Single‐centre Retrospective	Canada	IFD	Induction: 104 Salvage: 27	AML with *FLT3* mutation	Induction: Midostaurin or sorafenib^b^ (*n* = 27) Salvage: gilteritinib (*n* = 8), sorafenib^b^ (*n* = 1) or quizartinib (*n* = 1)	Induction: FCZ (89.4%) or micafungin (10.6%)	EORTC/MSG	Induction: Incidence of proven or probable IMI did not differ between patients receiving 3+7 induction therapy (*n* = 91/104) who did receive midostaurin (1/22 [4.5%]) and those who did not (3/69 [4.3%]). Equivalent data not given for sorafenib Salvage: Proven or probable IMI in 2 gilteritinib, 1 sorafenib and 0 quizartinib patients
Aleissa et al. [43]	Single‐centre Retrospective	USA	IFD	47	AML with *FLT3* mutation (*n* = 46)	Gilteritinib	Optional; given in 42.5%; most commonly FCZ	EORTC/MSG	Proven, probable or possible IFD in 3 (15%), 2 (10%) and 8 (40%), respectively, in patients receiving triazole therapy (*n* = 20) No detail on sites or pathogens 4 deaths due to fungal disease; 1 in a patient not receiving triazole prophylaxis (1/27; 3.7%); three in patients receiving triazole prophylaxis (3/20; 15%)
On et al. [44]	Multicentre Retrospective	USA	IFD	235	Newly diagnosed or r/r AML	VEN + HMA (DEC/AZA)	Optional; given in 67.2% of patients; most commonly PCZ, VCZ or ISAV	EORTC/MSG	Proven, probable or possible IFD in 7 (3%), 5 (2.1%) and 18 (7.7%), respectively; proven or probable IFD in 12/235 patients (5.1%) When specified, most common pathogens were *Aspergillus* (*n* = 4), *Candida* (*n* = 3) and *Mucor* (*n* = 2); species not identified in 21 cases Most commonly affected site was the lung (24/30 patients)
Zhang et al. [45]	Single‐centre Retrospective	USA	IFD	144	Newly diagnosed AML	VEN + AZA	Optional; given in 10 patients (6.9%); anidulafungin, FCZ and ISAV in 6, 4 and 1 patient, respectively^c^	EORTC/MSG	IFD in 25/144 patients (17%), of which 8/144 (5.6%) were proven or probable All proven or probable cases were consistent with invasive pulmonary aspergillosis One fatal case of proven invasive pulmonary aspergillosis No IFD cases occurred in patients receiving prophylaxis
Aldoss et al. [46]	Single‐centre Retrospective	USA	IFD	119	Newly diagnosed or r/r AML	VEN + HMA (DEC/AZA)	Optional; given in 79%; either azoles or micafungin	EORTC/MSG	Proven or probable IFD in 15/119 patients (12.6%) Most common pathogens were *Aspergillus* (*n* = 7) and *Mucor* (*n* = 5) Most commonly affected site was the lung (11/15 patients [73%]) 13 IFD cases occurred during prophylaxis
Chen et al. [47]	Single‐centre Retrospective	USA	IFD	131	Newly diagnosed AML	VEN + HMA (DEC/AZA)	Optional; given in 17%; most commonly FCZ	EORTC/MSG	Proven, probable or possible IFD in 17/131 patients (13.0%); proven or probable IFD in 4/131 patients (3.1%) Pathogens included *Candida parapsilosis*, *Candida krusei*, *Fusarium* spp. and *Aspergillus fumigatus* Most commonly affected site was the lung (14/17 patients) Two IFD cases (both possible) occurred in patients receiving prophylaxis
Wang et al. [48]	Single‐centre Retrospective	Taiwan	IFD	61	AML	VEN + AZA (*n* = 23)	Optional; given in 18/23 (78.3%); most commonly PCZ	EORTC/MSG	Proven or probable IFD in 6/23 patients (26%) Pathogens included *Aspergillus* for all probable cases and *Fusarium* spp. for one proven case Most commonly affected site was the lung (5/6 patients)
Lee et al. [49]	Single‐centre Retrospective	Republic of Korea	IFD	122	Newly diagnosed or r/r AML	VEN‐based combination therapy^d^	FCZ (98.1%), PCZ (1.9%)	EORTC/MSG	Proven or probable IFD in 22/122 patients (18%) *Aspergillus* in all IFDs Most commonly affected site was the lung (21/22 patients) Most cases occurred in patients receiving FCZ prophylaxis (19/22) 14/22 patients with IFDs died; IFDs accounted for 9/53 deaths overall (17.0%)
Rausch et al. [50]	Single‐centre Retrospective	USA	IFD	277	Newly diagnosed AML or HR‐MDS	VEN + HMA or low‐dose chemotherapy (*n* = 105)	PCZ (51%), VCZ (30%) or ISAV (19%) overall (*N* = 277; prophylaxis not reported for VEN + HMA or low‐dose chemotherapy group separately)	EORTC/MSG	Proven or probable IFD in 5 patients Included proven *Candida krusei* fungemia (*n* = 1); proven *Candida guillermondii* fungemia (*n* = 1); probable *Aspergillus* pneumonia (*n* = 2), probable disseminated *Aspergillus* spp.
Reynolds et al. [51]	Single‐centre Retrospective	Australia	IFD	99	Newly diagnosed or r/r AML (82%) or MDS (18%)	VEN + AZA (*n* = 54) VEN + low‐dose cytarabine (*n* = 33) VEN + cytarabine and daunorubicin (*n* = 9) VEN + other (*n* = 4)	PCZ	EORTC/MSG	Proven, probable or possible IFD in 6 patients receiving VEN + HMA and 3 patients receiving VEN + low‐dose cytarabine Pathogen not identified in most cases Most commonly affected site was the lung (8/9 patients); remaining case was proven disseminated *Lomentospora* The majority of patients with IFD had adequate PCZ levels during therapeutic drug monitoring
Wang et al. [52]	Single‐centre Retrospective	China	Efficacy/IFD	17	Newly diagnosed AML/high‐risk MDS or r/r AML	VEN + HMA	Azoles; VCZ (*n* = 3); PCZ (*n* = 14); VCZ→PCZ (*n* = 1)	NR	No cases of IFDs
Menna et al. [53]	Multicentre Prospective Proof‐of‐concept	Italy	PK/Efficacy	35	Newly diagnosed AML with *FLT3* mutation	Midostaurin	PCZ (54%) or micafungin (46%)	NR	IFDs in 1/19 patients receiving PCZ (5%) and 2/16 patients receiving micafungin (12.5%^e^; *p* = NS) No detail given on proven/probable/possible cases or pathogens
Perl et al. [54]	Multicentre Prospective Dose escalation Phase 1/2	Multinational—USA, France, Germany, Italy	Efficacy	265	r/r AML	Gilteritinib	NR	NR	Grade 3 fungal pneumonia in 11 patients (4%) No further details given
DiNardo et al. [55] DiNardo et al. [56]	Multicentre Prospective Dose escalation Phase 1b	USA	Efficacy	Initial study: *n* = 57 Expansion stage: *n* = 145	≥ 65 years with treatment‐naïve AML	VEN + HMA (DEC/AZA)	CYP3A inhibitor azole antifungals not permitted Initial study: non‐azole prophylaxis given in 53% Expansion study: echinocandins given in 46%	NR	Initial study: Grade 3 fungal infections in 2 patients Bronchopulmonary aspergillosis (*n* = 1); hepatic candidiasis (*n* = 1) Expansion study: Grade 3/4 fungal infections in 8% of patients One death due to fungal pneumonia
Chatzilygeroudi et al. [57]	Multicentre Retrospective	Greece	Efficacy	57	Treatment‐naïve AML	VEN + HMA (DEC/AZA; *n* = 40)	Optional; strong or moderate CYP3A4 inhibitors given in 22.5%; PJP prophylaxis given in 12.5% (*n* = 40)	NR	IFD in 3/40 patients (7.5%) No detail given on proven/probable/possible cases or pathogens
Sciumè et al. [58]	Single‐centre Retrospective	Italy	Efficacy	60	Newly diagnosed or r/r AML	VEN‐based combination therapy^f^	Optional for patients prior to November 2020; azole prophylaxis routine thereafter; given in 32 patients	NR	IFDs in 10/60 patients (17%) Included fungal pneumonia (*n* = 8), complicated urinary candidosis (*n* = 1) and systemic candidosis (*n* = 1) IFDs were more common among patients who did not versus did receive antifungal prophylaxis (26% vs. 6%)

Abbreviations: AML, acute myeloid leukaemia; AZA, azacitibine; CYP3A4, cytochrome P450 3A4; DEC, decitabine; EORTC/MSG, European Organization for Research and Treatment of Cancer Mycoses Study Group; FCZ, fluconazole; FLT3, Fms‐like tyrosine kinase 3; HMA, hypomethylating agent; HR, high‐risk; IFD, invasive fungal disease; IMI, invasive mould infection; ISAV, isavuconazole; MDS, myelodysplastic syndrome; NR, not reported; NS, not significant; PCZ, posaconazole; PJP, Pneumocystis jirovecii; PK, pharmacokinetics; r/r, relapsing/refractory; VCZ, voriconazole; VEN, venetoclax.

^a^ Shaded rows represent publications were not identified in the PubMed search but were identified from additional searches.

^b^ Sorafenib is not approved for AML in the United States or United Kingdom.

^c^ Patients may have received > 1 agent.

^d^ AZA, 4.9%; DEC, 92.6%; low‐dose cytarabine, 2.5%.

^e^ Reported as 17% in source paper; percentage re‐calculated as 12.5% (2/16).

^f^ AZA, 83%; DEC, 8%; cytarabine, 3%; no additional treatment, 5%.

Across papers, there were some differences in the terminology used to describe IFDs. Some reported the IFD syndrome (e.g. aspergillosis), while others reported the IFD pathogen (e.g., *Aspergillus fumigatus*). Some referred to, for example, [invasive] ‘candidosis’, while others referred to ‘candidiasis’. Some papers reported IFDs, while others focused on the subset of invasive mould infections (IMIs) only. IFDs are reported below using the terminology as given in the papers.

### 4.1 Studies and Analyses Examining Incidence of IFD

All but two of the IFD analyses stated that IFDs were defined according to the European Organization for Research and Treatment of Cancer Mycoses Study Group (EORTC/MSG) [39] criteria. In one study, IFD was defined as ‘microbiologically or radiologically documented’ [41]. In the other, criteria for defining IFD were not reported, though no cases of IFD were reported in this study [52].

#### 4.1.1 Prospective Studies

The Italian SEIFEM (Sorveglianza Epidemiologica InFezioni nelle EMopatie) group evaluated the incidence of IFD during induction, re‐induction and consolidation therapy with midostaurin + chemotherapy for AML with *FLT3* mutations. Prophylaxis was optional and was far more common during induction (106/114 patients [93%]) and re‐induction therapy (10/12 patients [83%]) than during consolidation therapy (73/160 chemotherapy courses [46%]; 79 patients). The type of prophylaxis varied according to treatment stage, with PCZ being most common during induction (48.2%) and an echinocandin being most common during re‐induction (50%) and consolidation therapy (16.9%). Proven, probable or possible IFD was reported in 23/114 patients (20.2%) and 4/12 patients (33.3%) during induction and re‐induction therapy, respectively. During consolidation therapy, seven cases of IFD were reported across a total of 160 courses (4.4%); all but one case occurred during the first consolidation course. In all three treatment stages, the most common pathogens were *Aspergillus* and *Candida* spp., and the most commonly affected site was the lung. IFD occurred with prophylaxis in 21/23, 4/4 and 0/7 patients during induction, re‐induction and consolidation therapy, respectively. No significant differences in IFD incidence were observed between prophylaxis agents. The authors noted that the rate of IFDs in patients receiving prophylaxis during the induction stage was higher than expected and warranted further investigation [29].

A single‐centre Phase 2 prospective study examined the incidence of IFD in patients receiving remission‐induction chemotherapy for AML (95%) or MDS (5%). Patients received either high‐intensity cytarabine‐containing remission‐induction chemotherapy (*n* = 36) or VEN and/or a FLT3 inhibitor (midostaurin or sorafenib [not currently approved in AML]; *n* = 32). All patients received ISAV prophylaxis. IFD was reported in five patients receiving VEN‐based remission‐induction chemotherapy (probable pulmonary aspergillosis in one patient and possible fungal pneumonia in four patients; percentages not reported). None of the patients receiving FLT3 inhibitor‐based remission‐induction chemotherapy developed IFD [40].

A multicentre prospective study reported on the incidence of infectious complications in patients receiving hypomethylating agents (HMA) (azacitidine or decitabine), with or without VEN. Prophylaxis was optional. Of the 67 microbiologically or radiologically documented pneumonia episodes reported (*n* = 62), 18 had a fungal aetiology (27%). Anti‐mould prophylaxis was given in 17/62 patients who developed pneumonia (27%). Ten of these 62 patients died due to pneumonia [41].

#### 4.1.2 Retrospective Analyses

##### 4.1.2.1 FLT3 Inhibitors

A single‐centre retrospective analysis assessed the incidence of IMI in patients with AML and *FLT3* mutations receiving induction or salvage chemotherapy. Patients received prophylaxis with either FCZ (89.4%) or micafungin (10.6%). Overall, 27/104 patients received FLT3 inhibitors during induction therapy (midostaurin: 85.2%; sorafenib: 14.8%). The most common induction regimen was 3+7 (cytarabine and idarubicin), given in 91/104 patients (87.5%). The incidence of proven or probable IMI did not differ between patients who received the 3+7 plus midostaurin regimen (1/22 [4.5%]) and those who received the 3+7 regimen without midostaurin (3/69 [4.3%]; *p* = not significant). Equivalent data were not given for sorafenib or for those who received alternative induction regimens. Of the 27 patients who received salvage treatment, 10 received FLT3 inhibitors (8 gilteritinib, 1 sorafenib and 1 quizartinib). Proven or probable IMI was reported in two, one and zero patients, respectively (percentages not reported). IFD species and sites were not reported according to the treatment [42].

In a second single‐centre retrospective analysis, 46 patients received gilteritinib for AML with *FLT3* mutation. Triazole therapy (most commonly FCZ) was given to 20 (42.5%) patients. Of those who received triazole therapy, proven, probable or possible IFD were reported in three (15%), two (10%) and eight (40%) patients, respectively; no detail was given on IFD sites or pathogens. There were four deaths due to fungal disease; one in a patient not receiving triazole prophylaxis (1/27; 3.7%) and three in patients receiving triazole prophylaxis (3/20; 15%) [43].

##### 4.1.2.2 VEN‐Based Combination Therapy

Nine retrospective analyses were reported on patients receiving VEN‐based combination therapy, most commonly VEN + HMA (Table 3). Antifungal prophylaxis was administered in the majority of patients in seven of the studies (67%–100%) [44, 46, 48, 49, 50, 51, 52] and in only a small proportion in the other two studies (6.9% in one and 17% in the other) [45, 47]. The incidence of proven or probable IFD (i.e. excluding possible IFD, where reported), ranged from 0% to 26%. *Aspergillus* tended to be the most common pathogen reported; other pathogens included *Candida, Mucor, Fusarium* and *Lomentospora*. The most commonly affected site in all studies was the lung [44, 45, 46, 47, 48, 49, 50, 51].

### 4.2 Efficacy Studies Reporting IFD as an AE

The majority of efficacy studies did not specify how IFDs were defined (e.g. EORTC/MSGERC criteria) or classified (proven/probable/possible). Most efficacy studies also did not specify which fungal pathogens were involved. It is assumed that the reported cases of IFDs in these studies are ‘probable’ or ‘possible’ rather than ‘proven’, except where stated. Details are reported below where available.

#### 4.2.1 FLT3 Inhibitors

Two multicentre studies examined the efficacy of FLT3 inhibitors in patients with AML. The first was a proof‐of‐concept study in which patients received remission‐induction chemotherapy plus midostaurin and antifungal prophylaxis (PCZ or micafungin) for newly diagnosed AML with *FLT3* mutations. IFDs were reported in 1/19 patients receiving PCZ (5%) and 2/16 patients receiving micafungin (12.5%; *p* = NS). No detail on the nature of the IFDs was given [53].

The second study was a Phase 1/2 dose escalation study in 265 patients receiving gilteritinib (prophylaxis use not specified) for r/r AML. A total of 191 patients had *FLT3* mutations at screening. Eleven cases of Grade 3 fungal pneumonia (4%) were reported [54].

#### 4.2.2 VEN‐Based Combination Therapy

Two studies examined the efficacy of VEN + HMA (either azacitidine or decitabine) in treatment‐naïve patients with AML. In the first study, a multicentre Phase 1b dose escalation study in patients aged ≥ 65 years, CYP3A inhibitor azole antifungals were not permitted. In the initial study (*n* = 57), non‐azole prophylaxis was given in 53% of patients; in the expansion stage of the study (*n* = 145), 46% of patients received echinocandins. Grade 3 fungal infections were reported in two patients during the initial study (bronchopulmonary aspergillosis [*n* = 1]; hepatic candidiasis [*n* = 1]), and Grade 3 or 4 fungal infections were reported in 8% of patients during the expansion stage, with one death due to IFD (fungal pneumonia) [55, 56].

In the second VEN + HMA study, prophylaxis was optional; in the VEN + HMA group (*n* = 40), strong or moderate CYP3A4 inhibitors were given in 22.5% of patients, and PJP prophylaxis was given in 12.5% of patients. IFDs were reported in 3/40 patients (7.5%); no detail was given on proven/probable/possible cases or pathogens [57].

The efficacy of VEN‐based combination therapy (HMAs in 92% of patients) was also examined among 60 patients with newly diagnosed or r/r AML. Prophylaxis was optional for patients prior to November 2020; a change in institution guidelines meant that azole prophylaxis became routine thereafter (*n* = 32). IFDs were reported in 10/60 patients (17%) and included fungal pneumonia (*n* = 8), complicated urinary candidosis (*n* = 1) and systemic candidosis (*n* = 1). IFDs were more common among patients who did not versus did receive antifungal prophylaxis (26% vs. 6%). All but one IFD occurred in patients who had never received or had interrupted, antifungal prophylaxis; the systemic candidosis case occurred while on ISAV. The authors noted that tumour response rates were not affected by use of azole prophylaxis, provided VEN dose reduction was performed in line with clinical standards [58].

### 4.3 Factors Predicting IFD Risk

Several studies have attempted to identify predictors of IFD risk in the AML population.

In one study, where one‐quarter of patients received an FLT3 inhibitor, increasing age and the presence of the *FLT3* internal tandem duplication mutations (as opposed to tyrosine kinase domain mutations) were associated with increased risk of proven or probable IMI during induction therapy (*p* < 0.05) in a bivariate analysis [42]. In a second analysis, age was shown to increase the risk of IFD during induction therapy with midostaurin + chemotherapy (*p* < 0.05) [29].

In patients receiving VEN‐based combination therapy, two studies failed to find any significant predictors of IFD risk [44, 45]. Three analyses suggested that the type of AML may affect IFD risk, with r/r AML having a higher risk than newly diagnosed AML [46, 51] and secondary/therapy‐related AML having a higher risk than de novo AML or AML with myelodysplasia‐related changes [49]. In one analysis, having fewer lines of therapy was associated with an increased risk of IFD (*p* < 0.05); as this is unexpected, the authors suggested it may be due to the inclusion of patients with MDS in the study [51]. Several other factors have been identified as potentially increasing the risk of IFD (all *p* < 0.05): lack of response to VEN + HMA therapy [46], non‐favourable European LeukemiaNet risk classification [48], presence of prolonged/chronic neutropenia [48, 51], poor patient physical fitness [47], presence of the *TP53* mutation [47] and (in contrast to the FLT3 inhibitor study above [42]) younger age [47].

## 5 Discussion

The widespread use of antifungal prophylaxis and difficulties in accurately capturing cases of IFD in the studies identified mean that estimating the true incidence and nature of IFD in patients receiving targeted treatments for AML is difficult. However, even with the use of prophylaxis, the incidence of reported IFD in many studies was above 8%, the threshold for when primary anti‐mould prophylaxis (though not IFD prophylaxis overall) has been recommended [33]. This suggests that, as recommended by the EHA [30], antifungal prophylaxis may be warranted in patients receiving targeted therapy for AML. In most trials and analyses, *Aspergillus* was the most commonly observed pathogen. This is in line with the worldwide epidemiological shift towards mould infections since prophylaxis with FCZ (which does not have activity against *Aspergillus* [59]) was introduced for cancer in the early 1990s [33]. In many of the studies we identified, the most commonly affected IFD site was the lung. Studies were not identified for all approved targeted agents, and the reported IFD prevalence varied greatly. This underscores the need for further investigation into the risk of IFD with all targeted agents, as well as the role of prophylaxis and underlying pathogen.

A limitation of our analysis is the fact that the level of IFD diagnosis detail reported differed significantly across publications. Regulatory bodies do not require pharmaceutical companies to provide details on IFDs that occur during AML trials, and infectious disease experts tend not to be involved in AML trial design [30]. As a result, many of the prospective efficacy studies identified in our search did not describe how IFDs were diagnosed or classified and reported only superficial IFD details. One therefore cannot assume that the reported cases in some of the studies are ‘proven’ or even ‘probable’ IFD cases as per the revised EORTC/MSGERC criteria [39]. Most of the publications we identified were retrospective IFD‐focused analyses; these analyses provided more detail on IFD diagnosis and classification than the prospective efficacy studies, though one should bear in mind the limitations and biases inherent in retrospective studies [60]. With infections forming a large part of AML patient care, these observations support the need for well‐designed prospective trials that focus on IFD incidence and provide detailed and accurate IFD data for patients with AML receiving targeted therapies.

The duration of antifungal prophylaxis varied between studies, as did the type of prophylaxis used. Azoles were a frequently used option, with FCZ and PCZ most commonly given. IFD development occurred during prophylaxis treatment for patients in a number of studies, yet the variety in prophylaxis treatments given and a lack of detail regarding the type of infection prevent concrete conclusions from being drawn. Guidelines on the use of targeted agents in AML do not provide specific recommendations on the duration of prophylaxis [30, 31], leaving physicians to rely on local protocols and personal experience. The ECIL guidelines ‘do not recommend PAP beyond remission‐induction chemotherapy, unless patients are to undergo re‐induction chemotherapy or intensified consolidation therapy’ – though these recommendations do not relate to targeted treatments specifically [34]. In one trial with midostaurin, prophylaxis use was far higher during induction therapy (93%) than during consolidation therapy (46%) – though, unexpectedly, IFD incidence was also far higher (20.2% vs. 4.4%) [29]. Such results may suggest that the midostaurin induction period carries particular risk, and antifungal prophylaxis strategies should be adapted accordingly. Other trials have reported a relatively long period of prophylaxis. In a trial with VEN + HMA, for example the median exposure to PCZ was 225 days [51]. Choosing to extend prophylaxis may be based on the advanced age and frequent comorbidities in this patient population [61].

In patients with AML undergoing chemotherapy, known risk factors for IFD include older age, prolonged/profound neutropenia and monocytopenia, use of purine analogues, the presence of indwelling catheters, alimentary mucositis, individual genetic susceptibilities and a lack of response to induction chemotherapy [33]. Several of the studies in our review analysed risk factors, but the results varied across the targeted agent in question. Larger epidemiological studies may be warranted to better understand predictors of IFD risk for each targeted agent, in order to better inform prophylaxis strategies.

Early and accurate diagnosis of IFD with identification of the causal species is vital for effective treatment of IFD, with late diagnosis equated with overtreatment and a poor prognosis [62, 63, 64]; however, limitations in current diagnostics may mean that up to 50% of IFDs are not diagnosed [63], suggesting that current reported rates of IFD may be underestimating the true extent of the problem [64]. Non‐specific symptoms can hinder the identification of fungal infection, leading to the potential for misattribution to a bacterial or viral cause and subsequent initiation of inappropriate treatment [64, 65, 66]. Even upon identification of a fungal cause, diagnostic certainty remains unclear in many instances. Per the EORTC/MSG guidance, infections are classified as ‘possible’, ‘probable’ or ‘proven’ depending on the microbiological or histopathological evidence available [39]; however, limitations in conventional diagnostic methods can make obtaining a ‘proven’ diagnosis unfeasible [65]. Indeed, in the studies reported here, a number of the cases of reported IFD were ‘possible’ rather than ‘proven’ or even ‘probable’, indicating that there was no positive corroborating mycological evidence, thus limiting the conclusions that can be drawn. Despite remaining the gold standard for the diagnosis of a number of IFDs, traditional histopathological and mycology culture–based techniques have a low sensitivity, slow turnaround times and often require invasive procedures to obtain tissue, which contribute to the diagnostic challenge [62, 67]. In addition, a number of fungal species are non‐culturable by conventional methods, requiring serological or molecular techniques for their identification [62]. The availability of non–culture‐based tests, such as galactomannan or β‐d‐glucan, has assisted with earlier diagnosis of IFD, though relying on a single mycological biomarker may result in an underestimation of disease incidence [66]. A combined approach to diagnostics, incorporating multiple antigen‐based techniques targeting serum and other samples, with conventional culture and polymerase chain reaction‐based assays, may hold the key to overcoming the challenges of individual diagnostic methods [66, 67].

In patients who develop IFD, treatment remains a challenge. The number of approved systemic antifungal treatments is limited, comprising four main classes as of September 2024: the polyenes, the azoles, the echinocandins and the pyrimidine analogue 5‐flucytosine [62, 68]. A number of fungal species are intrinsically resistant to one or more antifungal agents; indeed, the global emergence of multidrug‐resistant fungal species is an increasing cause for concern for severely immunocompromised patients, as well as a growing global threat to human health [62, 69]. Appropriate use of antifungal therapies is therefore essential; overuse can be costly, has the potential for toxicity and increases the risk of detrimental drug–drug interactions [64]. Interactions between those in the azole class and the CYP‐interacting targeted agents are a common cause of concern for physicians [30], and a number of contraindications and dose adjustments need to be considered [70]. In AML, a minimum dose reduction of at least 75% is recommended for the targeted agent VEN when used in combination with a strong CYP3A inhibitor such as PCZ [6]. The advent of new antifungal therapies may help to reduce the overreliance on existing antifungal classes and reduce the potential for drug–drug interactions and off‐target effects [68].

## 6 Conclusions

IFD is relatively common among patients with AML receiving targeted therapies, despite the use of prophylaxis. *Aspergillus* tends to be the most commonly reported pathogen, and most cases occur in the lungs. IFD data come largely from retrospective cohort reviews; prospective studies with detailed and accurate IFD reporting are required to better understand the incidence and nature of IFDs in this patient population. Large epidemiological studies may also be warranted to identify patients who are most at risk of developing IFDs with different targeted treatments; this information can then be used to ensure prophylaxis is implemented appropriately and where necessary. The development of national guidelines and collaboration with specialists outside of the field of haemato‐oncology (e.g. infectious disease and mycology experts) would also be of value, to ensure consistent application of prophylaxis strategies.

## Author Contributions

All authors have contributed equally to this article.

## Ethics Statement

The authors have nothing to report.

## Consent

The authors have nothing to report.

## Conflicts of Interest

Samir Agrawal: Research grants, advisory boards, speaker fees: AbbVie, Astellas, AstraZeneca, Gilead Science Ltd., Hikma, Janssen, Merck, Pfizer and Shionogi. Anjaneya Bapat: Honoraria: Gilead Sciences Ltd. and Napp/Mundipharma. Christopher P. Eades: Honoraria for educational work: Gilead Sciences Ltd., Mundipharma/Napp and Pfizer—fees paid to an educational fund, administered by the Mycology Reference Centre, Manchester. Shreyans Gandhi: Research grants/Honoraria or Advisory boards/Consultation: Alexion AstraZeneca, Celgene, Gilead Science Ltd., Jazz, Novartis, Pfizer and SOBI.

## Supporting information



Supporting Information

## Data Availability

Systematic review data are available upon request.

## References

[1] J. C. Valentine , C. O. Morrissey , M. A. Tacey , et al., “A Population‐Based Analysis of Invasive Fungal Disease in Haematology‐Oncology Patients Using Data Linkage of State‐Wide Registries and Administrative Databases: 2005–2016,” BMC Infectious Diseases 19, no. 1 (2019): 274. 30898090 10.1186/s12879-019-3901-y PMC6429824

[2] B. Rambaldi , D. Russo , and L. Pagano , “Defining Invasive Fungal Infection Risk in Hematological Malignancies: A New Tool for Clinical Practice,” Mediterranean Journal of Hematology and Infectious Diseases 9, no. 1 (2017): e2017012. 28101316 10.4084/MJHID.2017.012 PMC5224802

[3] J. D. Jenks , O. A. Cornely , S. C. A. Chen , G. R. Thompson , and M. Hoenigl , “Breakthrough Invasive Fungal Infections: Who Is at Risk?” Mycoses 63, no. 10 (2020): 1021–1032. 32744334 10.1111/myc.13148

[4] J. S. Little , Z. F. Weiss , and S. P. Hammond , “Invasive Fungal Infections and Targeted Therapies in Hematological Malignancies,” Journal of Fungi 7, no. 12 (2021): 1058. 34947040 10.3390/jof7121058 PMC8706272

[5] American Cancer Society , “Targeted Therapy Drugs for Acute Myeloid Leukemia (AML),” (2023), https://www.cancer.org/cancer/types/acute‐myeloid‐leukemia/treating/targeted‐therapy.html.

[6] AbbVie Deutschland GmbH Co. KG , “Venetoclax (Venclyxto) Summary of Product Characteristics,” (2024), https://www.ema.europa.eu/en/documents/product‐information/venclyxto‐epar‐product‐information_en.pdf.

[7] Les Laboratoires Servier , “Ivosidenib (Tibsovo) Summary of Product Characteristics,” (2024), https://www.ema.europa.eu/en/documents/product‐information/tibsovo‐epar‐product‐information_en.pdf.

[8] Astellas Pharma Europe B. V ., “Gilteritinib (Xosparta) Summary of Product Characteristics,” (2024), https://www.ema.europa.eu/en/documents/product‐information/xospata‐epar‐product‐information_en.pdf.

[9] Pfizer Europe MA EEIG , “Glasdegib (Daurismo) Summary of Product Characteristics,” (2022), https://www.ema.europa.eu/en/documents/product‐information/daurismo‐epar‐product‐information_en.pdf.

[10] Pfizer Europe MA EEIG , “Gemtuzumab ozogamicin (Mylotarg) Summary of Product Characteristics,” (2023), https://www.ema.europa.eu/en/documents/product‐information/mylotarg‐epar‐product‐information_en.pdf.

[11] Novartis Europharm Ltd ., “Midostaurin (Rydapt) Summary of Product Characteristics,” (2023), https://www.ema.europa.eu/en/documents/product‐information/rydapt‐epar‐product‐information_en.pdf.

[12] Daiichi Sankyo Europe GmbH , “Quizartinib (Vanflyta) Summary of Product Characteristics,” (2023), https://www.ema.europa.eu/en/documents/product‐information/vanflyta‐epar‐product‐information_en.pdf.

[13] K. D. Sahasrabudhe , M. Albrethsen , and A. S. Mims , “Emerging Small Molecular Inhibitors as Targeted Therapies for High‐Risk Acute Myeloid Leukemias,” Expert Review of Hematology 16, no. 9 (2023): 671–684. 37405412 10.1080/17474086.2023.2233701

[14] B. J. Wouters , “Targeting IDH1 and IDH2 Mutations in Acute Myeloid Leukemia: Emerging Options and Pending Questions,” Hemasphere 5, no. 6 (2021): E583. 34095766 10.1097/HS9.0000000000000583 PMC8171378

[15] Novartis Pharmaceuticals Corporation , “Midostaurin (Rydapt®)_USPI,” (2023), https://www.novartis.com/us‐en/sites/novartis_us/files/rydapt.pdf.

[16] Daiichi Sankyo Inc ., “Quizartinib (Vanflyta®)_USPI,” (2023), https://daiichisankyo.us/prescribing‐information‐portlet/getPIContent?productName=Vanflyta&inline=true.

[17] Astellas Pharma US Inc ., “Gilteritinib (Xosparta®)_USPI,” (2022), https://www.accessdata.fda.gov/drugsatfda_docs/label/2018/211349s000lbl.pdf.

[18] Agios Pharmaceuticals , “Ivosidenib (Tibsovo®)_USPI,” (2023), https://www.accessdata.fda.gov/drugsatfda_docs/label/2019/211192s001lbl.pdf.

[19] Rigel Pharmaceuticals Inc ., “Olutasidenib (RezlidhiaTM)_USPI,” (2022), https://www.rezlidhia.com/downloads/pdf/REZLIDHIA‐Full‐Prescribing‐Information.pdf.

[20] Bristol Myers Squibb , “Enasidenib (Idhifa®)_USPI,” (2023), https://packageinserts.bms.com/pi/pi_idhifa.pdf.

[21] Celgene Europe B.V ., “Enasidenib (Idhifa)_EMA Application Withdrawal,” (2019), https://www.ema.europa.eu/en/documents/withdrawal‐letter/withdrawal‐letter‐idhifa_en.pdf.

[22] Pfizer Inc ., “Gemtuzumab ozogamicin (MylotargTM)_USPI,” (2021), https://www.accessdata.fda.gov/drugsatfda_docs/label/2020/761060s003lbl.pdf.

[23] AbbVie Inc ., “Venetoclax (Venclexta®)_USPI,” (2022), https://www.rxabbvie.com/pdf/venclexta.pdf.

[24] Pfizer Inc ., “Glasdegib (DaurismoTM)_USPI,” (2023), https://www.accessdata.fda.gov/drugsatfda_docs/label/2023/210656s005lbl.pdf.

[25] V. Nasillo , I. Lagreca , D. Vallerini , et al., “BTK Inhibitors Impair Platelet‐Mediated Antifungal Activity,” Cells 11, no. 6 (2022): 1003. 35326454 10.3390/cells11061003 PMC8947638

[26] B. L. Lampson and M. S. Davids , “The Development and Current Use of BCL‐2 Inhibitors for the Treatment of Chronic Lymphocytic Leukemia,” Current Hematologic Malignancy Reports 12, no. 1 (2017): 11–19. 28116634 10.1007/s11899-017-0359-0 PMC5862560

[27] C. D. DiNardo , B. A. Jonas , V. Pullarkat , et al., “Azacitidine and Venetoclax in Previously Untreated Acute Myeloid Leukemia,” New England Journal of Medicine 383, no. 7 (2020): 617–629. 32786187 10.1056/NEJMoa2012971

[28] V. E. Kennedy and C. C. Smith , “FLT3 Mutations in Acute Myeloid Leukemia: Key Concepts and Emerging Controversies,” Frontiers in Oncology 10 (2020): 612880. 33425766 10.3389/fonc.2020.612880 PMC7787101

[29] C. Cattaneo , F. Marchesi , I. Terrenato , et al., “High Incidence of Invasive Fungal Diseases in Patients With FLT3‐Mutated AML Treated With Midostaurin: Results of a Multicenter Observational SEIFEM Study,” Journal of Fungi 8, no. 6 (2022): 583. 35736066 10.3390/jof8060583 PMC9224885

[30] J. Stemler , N. de Jonge , N. Skoetz , et al., “Antifungal Prophylaxis in Adult Patients With Acute Myeloid Leukaemia Treated With Novel Targeted Therapies: A Systematic Review and Expert Consensus Recommendation From the European Hematology Association,” Lancet Haematology 9, no. 5 (2022): e361–e373. 35483397 10.1016/S2352-3026(22)00073-4

[31] J. Stemler , S. C. Mellinghoff , Y. Khodamoradi , et al., “Primary Prophylaxis of Invasive Fungal Diseases in Patients With Haematological Malignancies: 2022 Update of the Recommendations of the Infectious Diseases Working Party (AGIHO) of the German Society for Haematology and Medical Oncology (DGHO),” Journal of Antimicrobial Chemotherapy 78, no. 8 (2023): 1813–1826. 37311136 10.1093/jac/dkad143 PMC10393896

[32] B. W. Teh , D. K. Yeoh , G. M. Haeusler , et al., “Consensus Guidelines for Antifungal Prophylaxis in Haematological Malignancy and Haemopoietic Stem Cell Transplantation, 2021,” Internal Medicine Journal 51, no. S7 (2021): 67–88. 34937140 10.1111/imj.15588

[33] J. A. Maertens , C. Girmenia , R. J. Brüggemann , et al., “European Guidelines for Primary Antifungal Prophylaxis in Adult Haematology Patients: Summary of the Updated Recommendations From the European Conference on Infections in Leukaemia,” Journal of Antimicrobial Chemotherapy 73, no. 12 (2018): 3221–3230. 30085172 10.1093/jac/dky286

[34] J. Maertens , S. Cesaro , G. Maschmeyer , et al., “ECIL Guidelines for Preventing *Pneumocystis jirovecii* Pneumonia in Patients With Haematological Malignancies and Stem Cell Transplant Recipients,” Journal of Antimicrobial Chemotherapy 71, no. 9 (2016): 2397–2404. 27550992 10.1093/jac/dkw157

[35] R. A. Taplitz , E. B. Kennedy , E. J. Bow , et al., “Antimicrobial Prophylaxis for Adult Patients With Cancer‐Related Immunosuppression: ASCO and IDSA Clinical Practice Guideline Update,” Journal of Clinical Oncology 36 (2018): 3043–3054. 30179565 10.1200/JCO.18.00374

[36] G. Redelman‐Sidi , O. Michielin , C. Cervera , et al., “ESCMID Study Group for Infections in Compromised Hosts (ESGICH) Consensus Document on the Safety of Targeted and Biological Therapies: An Infectious Diseases Perspective (Immune Checkpoint Inhibitors, Cell Adhesion Inhibitors, Sphingosine‐1‐Phosphate Receptor Modulators and Proteasome Inhibitors),” Clinical Microbiology and Infection 24, no. S2 (2018): S95–S107. 29427804 10.1016/j.cmi.2018.01.030 PMC5971148

[37] S. C. A. Chen , J. Perfect , A. L. Colombo , et al., “Global Guideline for the Diagnosis and Management of Rare Yeast Infections: An Initiative of the ECMM in Cooperation With ISHAM and ASM,” Lancet Infectious Diseases 21, no. 12 (2021): e375–e386. 34419208 10.1016/S1473-3099(21)00203-6

[38] S. E. Kidd , A. Abdolrasouli , and F. Hagen , “Fungal Nomenclature: Managing Change Is the Name of the Game,” Open Forum Infectious Diseases 10, no. 1 (2023): ofac559. 36632423 10.1093/ofid/ofac559 PMC9825814

[39] J. P. Donnelly , S. C. Chen , C. A. Kauffman , et al., “Revision and Update of the Consensus Definitions of Invasive Fungal Disease From the European Organization for Research and Treatment of Cancer and the Mycoses Study Group Education and Research Consortium,” Clinical Infectious Diseases 71, no. 6 (2020): 1367–1376. 31802125 10.1093/cid/ciz1008 PMC7486838

[40] P. Bose , D. McCue , S. Wurster , et al., “Isavuconazole as Primary Antifungal Prophylaxis in Patients With Acute Myeloid Leukemia or Myelodysplastic Syndrome: An Open‐Label, Prospective, Phase 2 Study,” Clinical Infectious Diseases 72, no. 10 (2021): 1755–1763. 32236406 10.1093/cid/ciaa358 PMC8130026

[41] A. Candoni , D. Lazzarotto , C. Papayannidis , et al., “Prospective Multicenter Study on Infectious Complications and Clinical Outcome of 230 Unfit Acute Myeloid Leukemia Patients Receiving First‐Line Therapy With Hypomethylating Agents Alone or in Combination With Venetoclax,” American Journal of Hematology 98, no. 4 (2023): E80–E83. 36651870 10.1002/ajh.26846

[42] P. Phoompoung , B. Henry , G. Daher‐Reyes , H. Sibai , and S. Husain , “Invasive Mold Infections in FLT3‐Mutated Acute Myeloid Leukemia,” Clinical Lymphoma, Myeloma & Leukemia 21, no. 5 (2021): e477–e482. 10.1016/j.clml.2020.10.014 33678591

[43] M. M. Aleissa , B. S. Alshehri , I. Gonzalez‐Bocco , et al., “Triazole Antifungal Use for Prophylaxis and Treatment of Invasive Fungal Diseases for Patients Receiving Gilteritinib,” Leukemia Research 108 (2021): 106610. 34048999 10.1016/j.leukres.2021.106610

[44] S. On , C. G. Rath , M. Lan , et al., “Characterisation of Infections in Patients With Acute Myeloid Leukaemia Receiving Venetoclax and a Hypomethylating Agent,” British Journal of Haematology 197, no. 1 (2022): 63–70. 35174480 10.1111/bjh.18051

[45] A. Zhang , T. Johnson , D. Abbott , et al., “Incidence of Invasive Fungal Infections in Patients With Previously Untreated Acute Myeloid Leukemia Receiving Venetoclax and Azacitidine,” Open Forum Infectious Diseases 9, no. 10 (2022): ofac486. 36225746 10.1093/ofid/ofac486 PMC9547523

[46] I. Aldoss , S. Dadwal , J. Zhang , et al., “Invasive Fungal Infections in Acute Myeloid Leukemia Treated With Venetoclax and Hypomethylating Agents,” Blood Advances 3, no. 23 (2019): 4043–4049. 31816059 10.1182/bloodadvances.2019000930 PMC6963254

[47] E. C. Chen , Y. Liu , C. E. Harris , et al., “Outcomes of Antifungal Prophylaxis for Newly Diagnosed AML Patients Treated With a Hypomethylating Agent and Venetoclax,” Leukemia & Lymphoma 63, no. 8 (2022): 1934–1941. 35289704 10.1080/10428194.2022.2047964 PMC9481998

[48] S. T. Wang , C. H. Chou , T. T. Chen , et al., “High Rate of Invasive Fungal Infections During Early Cycles of Azacitidine for Patients With Acute Myeloid Leukemia,” Frontiers in Cellular and Infection Microbiology 12 (2022): 1012334. 36530436 10.3389/fcimb.2022.1012334 PMC9748082

[49] R. Lee , S. Y. Cho , D. G. Lee , et al., “Infections of Venetoclax‐Based Chemotherapy in Acute Myeloid Leukemia: Rationale for Proper Antimicrobial Prophylaxis,” Cancers 13, no. 24 (2021): 6285. 34944903 10.3390/cancers13246285 PMC8699304

[50] C. R. Rausch , A. J. DiPippo , Y. Jiang , et al., “Comparison of Mold Active Triazoles as Primary Antifungal Prophylaxis in Patients With Newly Diagnosed Acute Myeloid Leukemia in the Era of Molecularly Targeted Therapies,” Clinical Infectious Diseases 75, no. 9 (2022): 1503–1510. 35325094 10.1093/cid/ciac230

[51] G. Reynolds , K. F. Urbancic , C. Y. Fong , and J. A. Trubiano , “Invasive Fungal Infection Following Venetoclax and Posaconazole Co‐Administration,” British Journal of Haematology 203, no. 4 (2023): 593–598. 37731068 10.1111/bjh.19116

[52] L. Wang , L. Gao , Z. Liang , X. Cen , H. Ren , and Y. Dong , “Efficacy and Safety of Coadministration of Venetoclax and Anti‐Fungal Agents Under Therapeutic Drug Monitor in Unfit Acute Myeloid Leukemia and High‐Risk Myelodysplastic Syndrome With Neutropenia: A Single‐Center Retrospective Study,” Leukemia & Lymphoma 65, no. 3 (2024): 353–362. 38069781 10.1080/10428194.2023.2290465

[53] P. Menna , F. Marchesi , C. Cattaneo , et al., “Posaconazole and Midostaurin in Patients With FLT3‐Mutated Acute Myeloid Leukemia: Pharmacokinetic Interactions and Clinical Facts in a Real Life Study,” Clinical and Translational Science 16, no. 10 (2023): 1876–1885. 37515369 10.1111/cts.13595 PMC10582652

[54] A. E. Perl , J. K. Altman , J. Cortes , et al., “Selective Inhibition of FLT3 by Gilteritinib in Relapsed or Refractory Acute Myeloid Leukaemia: A Multicentre, First‐in‐Human, Open‐Label, Phase 1–2 Study,” Lancet Oncology 18, no. 8 (2017): 1061–1075. 28645776 10.1016/S1470-2045(17)30416-3 PMC5572576

[55] C. D. DiNardo , K. W. Pratz , A. Letai , et al., “Safety and Preliminary Efficacy of Venetoclax With Decitabine or Azacitidine in Elderly Patients With Previously Untreated Acute Myeloid Leukaemia: A Non‐Randomised, Open‐label, Phase 1b Study,” Lancet Oncology 19, no. 2 (2018): 216–228. 29339097 10.1016/S1470-2045(18)30010-X

[56] C. D. DiNardo , K. Pratz , V. Pullarkat , et al., “Venetoclax Combined With Decitabine or Azacitidine in Treatment‐Naive, Elderly Patients With Acute Myeloid Leukemia,” Blood 133, no. 1 (2019): 7–17. 30361262 10.1182/blood-2018-08-868752 PMC6318429

[57] T. Chatzilygeroudi , I. Darmani , N. El Gkotmi , et al., “Real‐Life Multicenter Experience of Venetoclax in Combination With Hypomethylating Agents in Previously Untreated Adult Patients With Acute Myeloid Leukemia in Greece,” Journal of Clinical Medicine 13, no. 2 (2024): 584. 38276092 10.3390/jcm13020584 PMC10816211

[58] Sciumè M. , Bosi A. , Canzi M. , Ceparano G. , Serpenti F. , De Roberto P. , et al., “Real‐Life Monocentric Experience of Venetoclax‐Based Regimens for Acute Myeloid Leukemia,” Frontiers in Oncology 13 (2023): 1149298. 37051529 10.3389/fonc.2023.1149298 PMC10083332

[59] Sun Y. , Hu J. , Huang H. , Chen J. , Li J. , Ma J. , et al., “Fluconazole Is as Effective as Other Anti‐Mold Agents in Preventing Early Invasive Fungal Disease After Allogeneic Stem Cell Transplantation: Assessment of Antifungal Therapy in Haematological Disease in China,” Translational Cancer Research 9, no. 11 (2020): 6900–6911. 35117298 10.21037/tcr-19-2887 PMC8798361

[60] K. Talari and M. Goyal , “Retrospective Studies—Utility and Caveats,” Journal of the Royal College of Physicians of Edinburgh 50, no. 4 (2020): 398–402. 33469615 10.4997/JRCPE.2020.409

[61] E. Borlenghi , A. M. Roccaro , and C. Cattaneo , “Rethinking the Definition of ‘Less Intensive’ for Venetoclax‐Combining Regimens in Acute Myeloid Leukaemia Patients,” British Journal of Haematology 203, no. 4 (2023): 504–506. 37803499 10.1111/bjh.19138

[62] W. Fang , J. Wu , M. Cheng , et al., “Diagnosis of Invasive Fungal Infections: Challenges and Recent Developments,” Journal of Biomedical Science 30, no. 1 (2023): 42. 37337179 10.1186/s12929-023-00926-2 PMC10278348

[63] T. Satyanarayana , S. K. Deshmukh , and M. V. Deshpande , “Advancing Frontiers in Mycology & Mycotechnology: Basic and Applied Aspects of Fungi,” in Advancing Frontiers in Mycology and Mycotechnology: Basic and Applied Aspects of Fungi, ed. T. Satyanarayana , S. K. Deshmukh , and M. V. Deshpande (Springer, 2019).

[64] D. W. Denning , D. S. Perlin , E. G. Muldoon , et al., “Delivering on Antimicrobial Resistance Agenda Not Possible Without Improving Fungal Diagnostic Capabilities,” Emerging Infectious Diseases 23, no. 2 (2017): 177–183. 27997332 10.3201/eid2302.152042 PMC5324810

[65] M. M. Riwes and J. R. Wingard , “Diagnostic Methods for Invasive Fungal Diseases in Patients With Hematologic Malignancies,” Expert Review of Hematology 5, no. 6 (2012): 661–669. 23216596 10.1586/ehm.12.53 PMC3563387

[66] M. M. Ceesay , S. R. Desai , L. Berry , et al., “A Comprehensive Diagnostic Approach Using Galactomannan, Targeted β‐d‐Glucan, Baseline Computerized Tomography and Biopsy Yields a Significant Burden of Invasive Fungal Disease in at Risk Haematology Patients,” British Journal of Haematology 168, no. 2 (2015): 219–229. 25179933 10.1111/bjh.13114

[67] J. D. Jenks , J. P. Gangneux , I. S. Schwartz , et al., “Diagnosis of Breakthrough Fungal Infections in the Clinical Mycology Laboratory: An ECMM Consensus Statement,” Journal of Fungi 6, no. 4 (2020): 216. 33050598 10.3390/jof6040216 PMC7712958

[68] E. Puumala , S. Fallah , N. Robbins , and L. E. Cowen , “Advancements and Challenges in Antifungal Therapeutic Development,” Clinical Microbiology Reviews 37, no. 1 (2024): e0014223. 38294218 10.1128/cmr.00142-23 PMC10938895

[69] World Health Organization , “WHO Fungal Priority Pathogens List to Guide Research, Development and Public Health Action,” (WHO, 2022).

[70] J. Lindsay , B. W. Teh , K. Micklethwaite , and M. Slavin , “Azole Antifungals and New Targeted Therapies for Hematological Malignancy,” Current Opinion in Infectious Diseases 32, no. 6 (2019): 538–545. 31688198 10.1097/QCO.0000000000000611

