# Supplementary material for: Invasive Fungal Disease Associated With Targeted Agents for Acute Myeloid Leukaemia: A Systematic Review
Source: EJHaem. 2025 Mar 10;6(2):e1105. doi: 10.1002/jha2.1105 (PMC11892366; doi:10.1002/jha2.1105)
Supplement: Supplementary file 1 — Supporting Information [file JHA2-6-e1105-s001.docx]

**Supplementary Table 1. Overview of PubMed search string.**

| **Search number** | **Item** | **Search string** |
| --- | --- | --- |
| 1 | Malignancy | Acute leukaemia OR acute myeloid leukaemia OR acute myelogenous leukaemia OR “haematologic malignancy” OR “haematological malignancy” OR “haematologic neoplasm” OR “haematological neoplasm” OR bone marrow transplantation OR haematopoietic stem cell transplant OR autologous transplant OR autograft OR allogeneic transplant OR allograft |
| 2 | Targeted treatment | Midostaurin OR Quizartinib OR Gilteritinib OR Ivosidenib OR Olutasidenib OR Enasidenib OR Gemtuzumab OR Venetoclax OR Glasdegib OR “targeted treatment” OR “targeted agent” |
| 3 | Invasive fungal disease | Aspergillosis OR invasive aspergillosis OR Candidiasis OR invasive candidiasis OR Pichia kudriavzevii OR Nakaseomyces glabrata OR Meyerozyma guilliermondii OR Clavispora lusitaniae OR Diutina rugosa OR Cryptococcus OR Pneumocystis OR Blastomycosis OR Coccidioidomycosis OR Histoplasmosis OR Paracoccidioidomycosis OR Penicilliosis OR Sporotrichosis OR Mucormycosis OR Zygomycosis OR Trichosporon OR Malassezia OR Pseudozyma OR Moesziomyces OR Dirkmeia OR Rhodotorula OR Sporobolomyces OR Geotrichum OR Kodamaea OR Saccharomyces OR Saprochaete OR Magnusiomyces OR invasive fungal disease OR invasive fungal infection |
| 4 | Combined above searches | 1 AND 2 AND 3 |
